# The burden and distribution of cystic echinococcosis in Bhutan: a retrospective study

**DOI:** 10.1017/S0031182024001069

**Published:** 2024-11

**Authors:** Chador Tenzin, Tashi Dendup, P. R. Torgerson, Peter Deplazes, Sonam Zangmo, Chador Wangmo, Tsheten Tsheten, Tandin Zangpo

**Affiliations:** 1 Punakha General Hospital, Punakha, Bhutan; 2 Save the Children International, Bhutan Country Office, Chang Geydaphu, Thimphu, Bhutan; 3 Section of Veterinary Epidemiology, Vetsuisse Faculty, Winterthurerstrasse, Zurich, Switzerland; 4 Institute of Parasitology, Medical and Vetsuisse Faculty, University of Zurich, Zurich, Switzerland; 5 Department of Internal Medicine, Jigme Dorji Wangchuck National Referral Hospital, Thimphu, Bhutan; 6 National Center for Epidemiology and Population Health, College of Health and Medicine, Australian National University, Canberra, Australia; 7 Gidakom General Hospital, Thimphu, Bhutan

**Keywords:** Bhutan, burden of disease, disease surveillance, Echinococcosis, epidemiology, hydatid disease, risk factors

## Abstract

Cystic echinococcosis (CE), caused by *Echinococcus granulosus s.l.* is a neglected zoonosis posing a significant public health challenge. Little is known about human CE in Bhutan. This study was conducted to gain an understanding of the burden, distribution, and potential risk factors of CE in Bhutan. From January 2015 to December 2019 data from Jigme Dorji Wangchuck National Referral Hospital (JDWNRH) and 6 other district-level hospitals were reviewed. Descriptive statistics were used to summarize the data. DALYs and Poisson regression models were used to estimate the burden and explore the relationship between cases and possible risk factors. A total of 159 cases were recorded. Most cases (145) were admitted to the surgical ward and 14 cases were referred to India. The average annual incidence was 4.4 cases per 100 000 population. The burden of disease was estimated to be approximately 39 DALYs per year for treatment-seeking cases, or possibly 80 DALYs per year including non-treatment seeking cases. This translates to approximately to 5.2 DALYs and 10.2 per 100 000 per year respectively. The commonest sites of infection were the liver (78%) and lungs (13%). Most cases were treated with surgery (>82%), and more than 47% were admitted to the hospital for >4 days. Policy interventions targeting community engagement, awareness, education, high risk occupational groups, females, and those living in the endemic districts of the central and western regions may yield larger gains. More studies and the institution of a surveillance system can help better guide policy interventions.

## Graphical abstract

**Figure:**
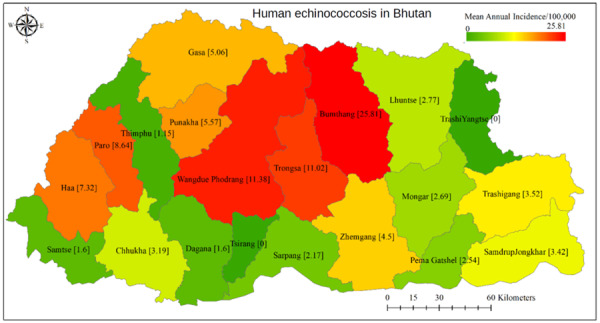

## Introduction

Cystic echinococcosis (CE) is a zoonotic disease with worldwide distribution, commonly in pastoral regions with a favourable environment for its life cycle (Higuita *et al*., 2016; Deplazes *et al*., 2017). Intermediate hosts such as sheep, cattle, goats, and other ungulates become infected by the ingestion of the parasite's eggs passed by definitive hosts such as wild and domestic dogs (Romig *et al*., 2017). Humans are dead-end intermediate hosts and do not perpetuate the parasite life cycle. CE is caused by the species complex of *E. granulosus sensu lato* (*s.l*.), which includes several genotypes that can differ in their pathogenicity and host range (Craig *et al*., 2007). The natural course of *E. granulosus* (*s.l*.) cyst growth in humans is not well understood and the rate at which the cysts grow was found highly variable depending on the age of the hosts, location of cysts, and other factors (Kern *et al*., 2017). However, most infections remain asymptomatic throughout life (Menghi *et al*., 2017). The presentation and severity of symptoms depend on the infection site, the size of the cyst, the organ involved, and its relation to surrounding structures and the cyst's wall integrity (Higuita *et al*., 2016; Menghi *et al*., 2017). CE in humans and animals is characterized by the expansive growth of echinococcus cysts in different parts of the body. In humans, the liver and lungs account for 69–75% and 17–22% of infection sites, respectively (Kern *et al*., 2017; Haleem *et al*., 2018).

As per the World Health Organization, around 1 million people are affected by CE (World Health Organization, 2020a). Worldwide, *E. granulosus sensu stricto* (*s.s.;* G1, G3) accounts for most of the human CE cases followed by genotypes of *E. canadensis* (G6, G7); all other species including *E. equinus* (G4), *E. ortleppi* (G5), *E. canadensis* (G8, G10) have only been found sporadically in CE patients (Deplazes *et al*., 2017). CE in humans is also endemic in South Asian countries like India, Bangladesh, and Nepal with the documented transmission in dogs and livestock (Deplazes *et al*., 2017). Globally, CE is responsible for an estimated 2225 deaths and 188 079 disability-adjusted life-years (DALYs) annually (Torgerson *et al*., 2015). Data from community studies suggest the prevalence can vary from a low of 1% to as high as 7% in endemic districts (Rojas *et al*., 2014). The control of this disease is challenging owing to the absence of acute symptoms and long incubation period, and the treatment is also complicated – often requiring admission to a hospital, as well as prolonged hospitalization (Tamarozzi *et al*., 2020).

In Bhutan, around 65% of the population lives in rural areas depending on subsistence farming and almost all the rural household own cattle (National Statistics Bureau, 2018). Many rural residents raise dogs mainly to protect their homes and farms, and similarly owning dogs as pets has increasingly become common in urban Bhutan. For instance, around 40% of the rural residents owned a pet dog, while about 21% of urban households in major urban centres owned pet dogs (Humane Society International, 2018). The stray and/or community dog population is also high throughout the country, especially in urban areas (Rinzin *et al*., 2016).

A molecular epidemiology study in Bhutan detected the occurrence of *E. granulosus s.s.* (G1) and *E. ortleppi* (G5), suggesting the potential role of dogs, cattle, sheep, and yaks in the local transmission of CE in Bhutan (Thapa *et al*., 2017). Recently, a subsequent study investigated the environmental contamination with *Echinococcus* eggs in populated areas of all districts documenting parasite transmission in several districts (Sharma *et al*., 2021). These factors such as living in rural areas, dog ownership, feeding dogs, slaughtering at home, being a farmer, and unsafe drinking water sources were shown to be important determinants of CE (Wang *et al*., 2014; Acosta-Jamett *et al*., 2014; Possenti *et al*., 2016; Yuan *et al*., 2017). The existence of these favourable conditions and risk factors suggests that a large proportion of Bhutan's population is at risk for CE. For instance, CE is commonly encountered in Bumthang and other districts where much of the population participate in activities involving livestock. In addition, recently, the contamination of *Echinococcus* eggs in environmental dog fecal samples have been documented (Sharma *et al*., 2021).

Epidemiological data on the burden and risk factors of human CE, which is essential to develop cost-effective interventions (Kebede *et al*., 2009) is scarce in Bhutan. A study that examined the clinical profiles of patients with CE in 2017 showed a longer treatment duration and favourable surgical treatment outcomes (Kelzang *et al*., 2019). Although there is a Zoonotic Disease Control Program under the Department of Public Health of the Ministry of Health, prevention and control of echinococcosis seem to have received less attention thus far. This could be mainly attributable to the lack of epidemiological data and consequently the perceived low burden and severity. While not much is known about it in the past, increasing cases were being detected with the recent introduction of imaging services such as ultrasonography (US) in district hospitals and computerized tomography (CT) scan in regional and other hospitals. This retrospective review of records was conducted to gain a preliminary understanding of the burden and distribution of CE and the potential risk factors. These findings can help the related health programmes to design prevention policies and prioritize future research areas.

## Materials and methods

This study retrospectively reviewed the hospital records of CE cases detected and recorded in the 7 hospitals of Bhutan including the Jigme Dorji Wangchuck National Referral Hospital (JDWNRH), the national referral hospital of Bhutan. The other 6 district-level hospitals were Wangdicholing (in Bumthang district), Yebilaptsa (in Zhemgang district), Trongsa, Wangdue Phodrang, Punakha, and Gasa. These hospitals were visited from 21^st^ May 2022 to 20^th^ June 2022 by 1 of the investigators. Data were obtained only from the ultrasonography (US) units of district hospitals, while the records of the surgical department, radiology department, medical records section, and ex-country referral section of JDWNRH were extracted and reviewed. For 2 hospitals, namely Gasa and Yebilaptsa (in Zhemgang district), data were only available for recent years since US services were instituted only from 2017 onwards. Due to the lack of advanced diagnostic facilities and clinical expertise, CE cases were not managed at the district level and regional referral hospitals of Bhutan. The probable cases from all districts requiring further investigations including surgical intervention are usually referred to and managed at the JDWNRH until recent installation of CT scan in regional and other hospitals. Thus, the records mainly from JDWNRH were analysed in this study. Data from the 6 districts were also analysed to supplement the main analysis (Table 1). Using data from JDWNRH for the main analysis can help prevent the potential double counting of cases given that there is no formal recording and reporting system for CE currently.

**Table 1.** Total number of cystic echinococcosis cases detected by US and total human and domestic animal population in the 6 districts where field visit was made

Districts	Cases, *N* = 92 (*N*, %)^1^	Pop^2^	Ani: Hu Ratio^3^	Domestic animal population (in number)^4^				
				Cattle	Dogs	Yaks	Sheep	Goats
Bumthang	36 (39.13)	17 820	0.91	11 311	1,017	3,917	127	9
Punakha	16 (17.39)	28 750	0.43	11 045	1,394	0	146	208
Trongsa	6 (6.52)	19 960	0.62	11 583	544	226	132	92
Zhemgang^5^	1 (1.09)	17 763	0.71	12 110	581	0	0	37
Wangdue^6^	32 (34.78)	42 186	0.68	23 306	1,152	4,115	948	88
Gasa^5^	1 (1.09)	3,952	1.99	1,163	193	6,527	0	2

US, ultrasonography.

1 Cases detected by the US in the six districts from 2015–2019.

2 Population, 2017 National Census, Bhutan.

3 Domestic animal (cattle, dogs, and yaks) to human population ratio.

4 Source: Livestock statistics, 2017, Ministry of Agriculture and Forest, Bhutan.

5 The data was only available for the few recent years since US services were instituted only from 2017 onwards.

6 Wangdue Phodrang.

A case of CE is defined as any patient with a clinical or epidemiological history and imaging findings or serology positive for CE on 2 tests with either; (1) demonstration of protoscoleces or their components, using direct microscopy or molecular tools in the cyst contents aspirated by percutaneous puncture or at surgery, or (2) changes in US appearance, e. g., detachment of the parasitic cyst in a CE1 cyst, thus moving to a CE3a stage, or solidification of a CE2 or CE3b, thus changing to a CE4 stage, after administration of benzimidazoles (for at least 3 months) or spontaneously (Kern *et al*., 2017). US cystic liver images are classified using the World Health Organization Informal Working Group on Echinococcus (WHO-IWGE) classification criteria. The diagnoses of the CE cases included in this study were based on the US and CT scans, as serology is not performed routinely in comparable tests. The imaging results, however, are currently not classified using the WHO-IWGE in the health facilities of Bhutan. Only cases confirmed following referral and treatment (usually surgical treatment) were included in the study.

The information collected from the hospital records included age, gender, permanent address, occupation, year of case registration, site of infection, total number of males and females scanned with US in JDWNRH, and type of treatment provided, and treatment days in the hospital. Information on the number of cattle, dogs, yaks, sheep, and goats in each of the districts was obtained from the 2017 Livestock Statistics, Ministry of Agriculture and Forest (Ministry of Agriculture, 2017). Information on the human population was obtained from the Population and Housing Census of Bhutan Report 2017 (National Statistical Bureau, 2017) and the Statistical Year Books published annually by the National Statistics Bureau of Bhutan. These data sets were used to estimate the incidence rates of CE for each district, different age groups and gender. Exact binominal 95% confidence interval was calculated for means of binomial variables, according to the method of Clopper and Pearson (Clopper and Pearson, 1934).

The burden of disease was estimated using the Disability Adjusted Life Year (DALY). The DALY has 2 components, the years of life lost due to premature mortality (YLLs) and the years lived with disability (YLDs). DALY = YLL + YLD (Devleesschauwer *et al*., 2014). YLL is difference between the age of death in fatal cases and the assumed residual life expectancy. The life table used was that recommended by the World Health Organisation (World Health Organization 2020b). The case fatality rate associated with CE for cases seeking treatment was assumed to be 2%. It was also assumed that morbidity associated with CE that is treated was for a period of approximately 2 years. The respected disability weights (DW) and standard deviations for hepatic echinococcosis was 0.123 (0.02), for pulmonary echinococcosis was 0.192 (0.132) and for other or multiple organ involvement 0.221 (0.04) (Salomon *et al*., 2012; Torgerson *et al*., 2015). Uncertainty in the incidence of CE was modelled using a gamma distribution. Uncertainty in the proportion of cases in each age group was modelled using a Dirichlet distribution. Uncertainty in the disability weights was modelled using the mean and standard deviations of the DW. Based on ultrasound population studies, it has been estimated that for every case of CE that is treated, there may be 100 cases that remain undiagnosed and untreated (Budke *et al*., 2013). As an additional scenario it was assumed that such cases would have a negligible case fatality rate and a low disability weight of 0.012 with a duration of 10 years.

A Poisson regression model was used to explore any relationship between numbers of reported cases of CE and population density of livestock species and dogs in the districts of Bhutan. All analysis was undertaken in R (R Core Team, 2023) and the code is provided as additional information.

## Results

### The burden of cystic echinococcosis

Based on the records maintained with the JDWNRH, a total of 145 CE cases were recorded in the surgical department and another 14 cases were referred to other hospitals outside the country (e.g., India) in the 5-year period of 2015–2019. This gave a crude case notification rate of 22 hospitalized CE cases per 100 000 population or an average annual incidence of 4.4 CE cases per 100 000 inhabitants.

The number of cases recorded in 2016 and 2017 with 40 cases each year resulting in an annual incidence of 5.42 cases per 100 000 inhabitants. This was not significantly different from the numbers of cases diagnosed in 2015, 2018, and 2019 (Table 2) with annual incidences of 3.43, 3.71, and 3.40 per 100 000 population, respectively.

**Table 2.** Number of cystic echinococcosis (CE) cases and annual incidences per 100 000 inhabitants recorded in Jigme Dorji Wangchuck National Referral Hospital (JDWNRH) from 2015 to 2019

Year	CE cases	Total population	Incidence	95%–CI
2015	25	727 876	3.43	2.23–5.10
2016	40	736 708	5.42	3.86–7.38
2017	40	745 563	5.36	3.81–7.32
2018	28	754 388	3.71	2.46–5.38
2019	26	763 092	3.40	2.21–4.98

**Figure 1.**
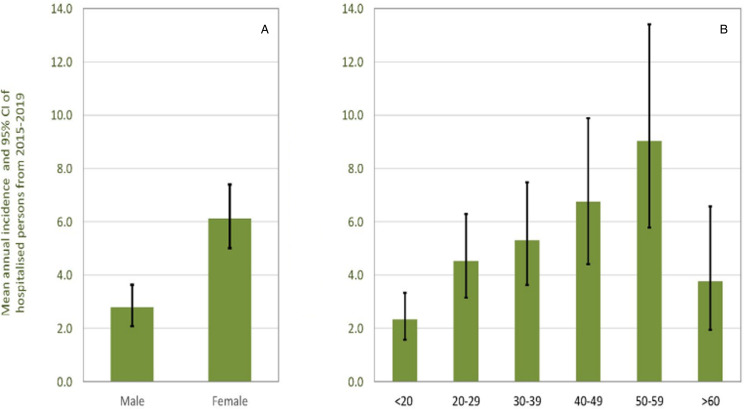
Annual incidences per 100 000 inhabitants (A) per gender and (B) age class with a diagnosis of cystic echinococcosis within 5 years (2015–2019). Error bars: exact binomial 95% confidence intervals. Population by age group: <20 = 257703, 20–29 = 154695, 30–39 = 120729, 40–49 = 77028, 50–59 = 53 215, ≥60 = 63 775.

Table 3 shows that a majority (66.67%) of the cases detected and recorded in JDWNRH were females and farmers. The majority of cases were hepatic echinococcosis. Correspondingly, the annual incidence in females was significantly higher than in males (6.11 *vs* 2.79, *P* < 0.001) (Fig. 1A).

**Table 3.** Distribution of cystic echinococcosis cases recorded in JDWNRH by sociodemographic and clinical characteristics

Variable	Categories	Cases *N* (%)
*Sociodemographic*	*N* = 159	
Age	<20	30 (18.87)
	20–29	35 (22.01)
	30–39	32 (20.13)
	40–49	26 (16.35)
	50–59	24 (15.09)
	≥60	12 (7.55)
Gender	Female	106 (66.67)
	Male	53 (33.33)
Occupation	Farmer	81 (50.94)
	Student^1^	32 (20.13)
	Business	8 (5.03)
	Employed^2^	21 (13.21)
	Unknown	17 (10.69)
Region	Western	58 (36.48)
	Central	69 (43.40)
	Eastern	24 (15.09)
	Unknown	8 (5.03)
*Clinical*		
Site of infection	Liver	124 (77.98)
	Lungs	21 (13.21)
	Both liver and lungs	4 (2.52)
	Intra-abdomen & Pelvis	6 (3.77)
	Spleen	2 (1.26)
	Liver & Spleen	1 (0.63)
	Unknown	1 (0.63)
Length of stay in hospital	<5 days	59 (37.11)
	5–7 days	51 (32.08)
	8–10 days	11 (6.92)
	>10 days	14 (8.81)
	Unknown	24 (15.09)
Treatment type	Surgery	131 (82.39)
	Conservative management	9 (5.66)
	Refused surgery	1 (0.63)
	Unknown^3^	18 (11.32)

JDWNRH, Jigme Dorji Wangchuck National Referral Hospital; *N*, number.

1 Student: includes student, monks, and nuns.

2 Employed: includes uniform personnel, government, and private employees.

3 Includes the 14 cases referred out of the country.

The most common sites of infection were the liver (78%), followed by the lungs (13%) (Table 3). However, specific information on whether cases were diagnosed based on thoracic x-ray (chest x-ray) was not available. Infection of other sites such as the spleen, pelvis, and retroperitoneum accounted for a very small share. Four patients had infections in both the liver and lungs. Infections of sites such as the brain, heart, bones, and other sites were not recorded in the surgical department in the last 5 years. The majority (~48%) of the patients were admitted to the hospital for more than 5 days for management and most (82%) received surgical treatment (Table 3). About 6% of the patients were managed conservatively. The youngest patient that was operated on was 8 years old and the oldest was 85 years old. Fourteen patients with CE in the lungs or with some other complications were referred out of the country for treatment.

The individual risk for a diagnosis with CE increased strongly with increasing age and the estimated annual incidence was significantly lower in the age group <20 years (2.33) than in adults from 30–39, 40–49, and 50–59 years 5.30, 6.75 and 9.02, respectively. (Fig. 1B). Surprisingly the estimated annual incidence decreased for people >60 years (3.76), which is even lower than for the age group 20–29 (4.53).

The burden of disease, using the data on the clinical location of the cysts, the demographics of the reported cases (Table 3) and the reported incidence on CE was 41 DALYs per year (95% uncertainty intervals 32–51 DALYs). The additional burden of disease that may be attributed to cases not seeking treatment was estimated at 39 DALYs per year (95% uncertainty intervals 33–45 DALYs). The total burden of disease attributed to treatment seeking and non-treatment seeking cases may be estimated at 80 DALYs per year (95% uncertainty interval of 66–96 DALYs). This equates to 10.2 DALYs per 100 000 per year (95% uncertainty intervals 8.4 to 12.2 DALYs per 100 000 per year).

### Geographical distribution

**Figure 2.**
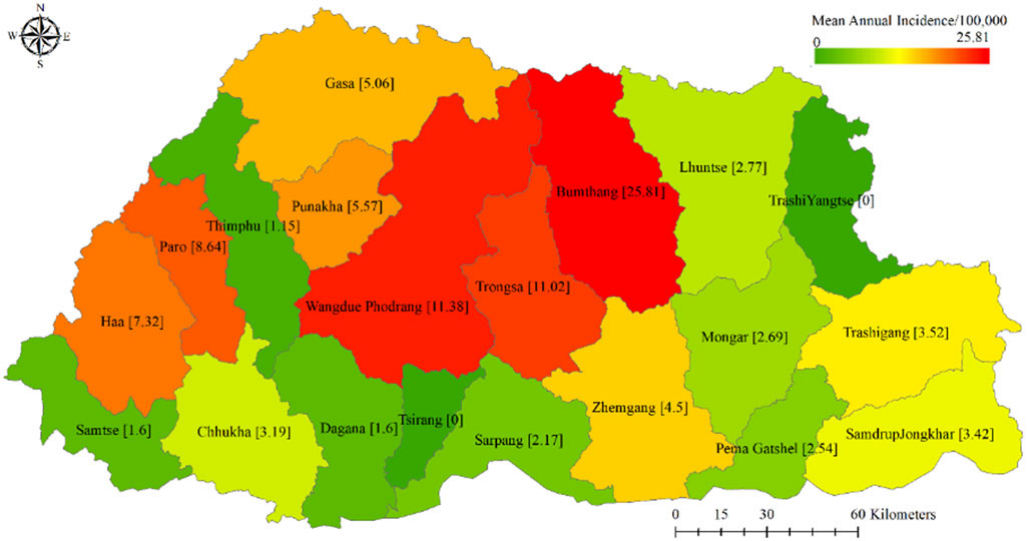
Annual incidences of cystic echinococcosis by districts in Bhutan per 100 000 population (2015–2019).

Although there were at least one or more cases from almost all the districts, the highest number of cases detected were from Wangdue Phodrang (15.89%, *N* = 24) followed by Bumthang (15.23%, *N* = 23), Paro (13.25%, *N* = 20), Trongsa (7.28%, *N* = 11), and Chhukha (7.28%, *N* = 11) (Table 4). The mean annual incidence was highest in Bumthang, Wangdue Phodrang, Trongsa, and Paro with the rates of 25.8, 11.38, 11.02, and 8.64 cases per 100 000 population, respectively. Low numbers of cases were reported from Gasa with only one case, and Dagana and Lhuntse reported two cases each. No cases were reported from Tashi Yangtse and Tsirang districts during this period. When grouped into regions, the central region (43.40%) had the largest proportion of cases notified followed by western (36.48%) and eastern (15.09%) regions (Table 3). The regional annual mean case notification rate was also significantly higher in the central region than in the western and the eastern regions (annual incidence rates: 7.2, 3.2, and 2.8 cases per 100 000 population). The review of US records in 6 districts also revealed that most cases detected were from Bumthang and Wangdue Phodrang (Table 4). The geographical distribution of incidence is also illustrated by Fig. 2. The distribution of CE by districts partly corresponds with the population of cattle and dogs in these districts (Table 1). The Poisson regression model was unable to demonstrate a significant association between livestock or dog populations and the district incidence of CE.

**Table 4.** Hospitalized cystic echinococcosis cases (2015–2019) and annual incidences by districts and regions in Bhutan

	*N* (population)	*N* (cases)/5 years	Rate (%)	95% CI	Mean annual incidence/100 000
Districts					
Bumthang	17 820	23 (15.23)	0.13	0.082–0.194	25.81
Chhukha	68 966	11 (7.28)	0.02	0.008–0.029	3.19
Dagana	24 965	2 (1.32)	0.01	0.001–0.029	1.6
Gasa	3952	1 (0.66)	0.03	0.001–0.141	5.06
Haa	13 655	5 (3.31)	0.04	0.012–0.086	7.32
Lhuntse	14 437	2 (1.32)	0.01	0.002–0.050	2.77
Mongar	37 150	5 (3.31)	0.01	0.004–0.031	2.69
Paro	46 316	20 (13.25)	0.04	0.026–0.067	8.64
Pema Gatshel	23 632	3 (1.99)	0.01	0.003–0.037	2.54
Punakha	28 740	8 (5.30)	0.03	0.012–0.055	5.57
SamdrupJongkhar	35 079	6 (3.97)	0.02	0.006–0.037	3.42
Samtse	62 590	5 (3.31)	0.01	0.003–0.019	1.6
Sarpang	46 004	5 (3.31)	0.01	0.004–0.025	2.17
Thimphu	138 736	8 (5.30)	0.01	0.003–0.011	1.15
Trashigang	45 518	8 (5.30)	0.02	0.008–0.035	3.52
TrashiYangtse	17 300	0	0	0.000–0.017	0
Trongsa	19 960	11 (7.28)	0.06	0.028–0.099	11.02
Tsirang	22 376	0	0	0.000–0.013	0
Wangdue Phodrang	42 186	24 (15.89)	0.06	0.037–0.085	11.38
Zhemgang	17 763	4 (2.65)	0.02	0.006–0.058	4.5
Regions					
East	173 116	24 (15.89)	0.01	0.009–0.021	2.77
Central	191 074	69 (45.70)	0.04	0.028–0.046	7.22
Western	362 955	58 (38.41)	0.02	0.012–0.021	3.2
Total	727 145	151^1^			

1 Eight of the 159 cases did not have information of the district of origin.

## Discussion

The incidence for hospitalized patients, however, may not represent the actual burden of the disease in the country as documented in other endemic areas (Possenti *et al*., 2016). For instance, those that are asymptomatic, those with mild symptoms, and those who do not require health services will be missed in the hospital records. Furthermore, many diagnosed cases may also have been managed conservatively on an outpatient basis especially those with uncomplicated inactive cysts on watch and wait for management plan in other referral hospitals. Likewise, some cases could be possibly managed at the district hospitals. Additionally, the sonographers may have also potentially missed infection in the early stages and infection of rare sites in asymptomatic individuals. Therefore, prevalence surveys are needed to provide more precise estimates of the burden of CE. A standardized recording and reporting system, that is currently lacking, can be useful in providing timely and more accurate information regarding case detection and management.

The data from the study was from 5 years previously. However, it is the most up-to-date information available. Since 2021 there has been a major restructuring of health infrastructure. Previous to 2021 CE cases were referred to the National Referral Hospital for treatment and thus data from this hospital represents an estimate of the national incidence of CE. Subsequently, many more cases have been treated at upgraded local facilities. Due to resource constraints resulting from limited funding, and disruption due to COVID-19 pandemic it has not been possible to investigate the records of these additional centres.

The gender distribution with the majority being females is consistent with other reports which usually demonstrate a higher incidence in females (Budke *et al*., 2013). The difference in gender roles may help explain this finding. Women may be more involved in looking after the cattle and dogs, gardening, and preparing meals by which their exposure risk is increased as compared to males. Traditionally, Bhutanese women are tasked with household chores and such a role of women in the family still exists, especially in rural Bhutan. It may be also that females are more often investigated using US. For instance, around 62% of the total US performed in JDWNRH in the last 5 years were females.

Although rates are usually greater among older adults people given the long incubation period, the largest proportion (~42%) of the total cases have been registered in the age group 20–39 due to demographic reasons (37% of the total population) (Table 3). Generally, young adults might be the ones who attend to household activities more often, including livestock-related work. Many old adults remain asymptomatic throughout life and remain undiagnosed, while some may not be fit for surgery due to their age and other associated comorbidities (Budke *et al*., 2013). This could have potentially led to the low number of cases reported among older adults and the elderly in hospital-based records.

Of the total number of cases, most were farmers (50.94%), followed by students (20.13%) (Table 3). This is expected as farmers, who represent much of the rural population, come in direct contact with dogs and the environment contaminated by infectious hosts. A review study showed that herdsmen were the most at risk of CE in the Qinghai-Tibet Plateau, China (Wang *et al*., 2014). Children love touching animals and playing in the open field; thus, they are more likely to encounter dogs and contaminated environments. The risk is even higher for children living in villages given the easy access to open fields to play and work, higher stray dog population, and poor sanitation and access to safe drinking water (Yuan *et al*., 2017).

In several districts, contamination of the environment with *E. ortleppi* and or *E. granulosus* s.s. eggs in dog fecal samples have previously been documented (Wangdue Phodrang 18 of 143, Bumthang 3 of 50, Paro 5 of 28, and Trongsa 1 of 24 samples contained *Echinococcus* eggs) (Sharma *et al*., 2021). Interestingly, a study that collected fecal samples from all 20 districts selecting areas with a high population of owned and free roaming/stray dogs found the environmental contamination with *Echinococcus* eggs were also very low in these districts with only two out of 37 and one out of 23 samples investigated contained echinococcus eggs respectively (Sharma *et al*., 2021).

The review of US records revealed that most cases detected were from Bumthang and Wangdue Phodrang (Table 4). The greater number of cattle and dog population in these districts was hypothesized to provide some explanation. Similarly, the greater domestic animal (cattle, dogs, and yaks) to human population ratio in Bumthang compared to other districts including Wangdue Phodrang also render some support for the higher number of cases notified from Bumthang. However, the Poisson regression model was unable to demonstrate a significant association between livestock or dog populations and the district incidence of CE.

While it is likely that rural residents in these districts are more likely to be at a heightened risk, information on rural-urban residences were not available in the records maintained with the hospitals. For instance, those in rural areas may be exposed through drinking water contaminated with the feces of infected definitive hosts (Wang *et al*., 2014; Yuan *et al*., 2017). Lower case notification from the eastern region may also be partly due to poor access to health facilities than the central and western regions.

The most common sites of infection were the liver followed by the lungs (Table 3). Infection of sites other than the liver of lungs were seldom reported. The lack of reports of infections of the brain, heart, bones, and other sites were not recorded could be because the management of infection of cases might have been dealt with by the specialized department. Alternatively they might have been directly referred out of the country (India) without admission to the surgical ward. For instance, a few cases of cardiac hydatid cysts were reported in Bhutan (Giri *et al*., 2020) but no records were available in the surgical department. Similarly, a case of CE in the spine was also managed by the orthopedic department of JDWNRH (Dr K. Wangdi, Spine Surgeon, email communication, September 06, 2022).

These data suggest that CE also contributes to the increasing health care cost in Bhutan. Interventions aimed to detect and manage cases early would not only prevent complications but also reduce associated treatment and referral costs. However, the length of stay and treatment type was not known for 24 and 18 cases respectively, and information on treatment outcome was not available in the records.

For comparison, we have illustrated other national of subnational studies on the burden of CE in terms of annual numbers of DALYs per 100 000 population (Table 5). Unfortunately, not all studies were standardized. Some of the earlier studies used age weighting (where a year of life lost in a young adult is given greater weighting to one lost as a child or an older adult) and discounting. These have now been abandoned so that more recent studies weight age equally and do not discount into the future. Most studies only estimated the burden of disease due to reported cases, and these would be directly comparable to the lower measure of reported cases in Bhutan. For example from Italy (Piseddu *et al*., 2017), which suggests a similar annual burden incidence per 100 000 population compared to Bhutan. However, it is likely that as Italy is an upper income country, cases are more likely to receive treatment compared to a lower- or middle-income country such as Bhutan. The Qinghai-Tibet Plateau (China) has the highest estimated incidence of DALYs. The data was obtained from a number of recent published reports on mass surveillance and 10 government data bases and is the unlikely to suffer from underreporting. This region of China is one of the most highly endemic regions in the world for CE of China and illustrates an upper limit to the likely burden of disease due to CE.

**Table 5.** Comparison with other studies that have estimated DALYs due to cystic echinococcosis on a national or subnational scale

Country or District	Estimated annual DALYs per 100 000 population	Study	Notes
Bhutan	10.2 (8.4–12.2) 5.2 (4.0–6.6)	Present study	Estimated all cases Treated cases
Kyrgyzstan	44 (22–74)	Counotte *et al*. (2016)	Treated cases only, corrected for under reporting
Italy	5.3	Piseddu *et al*. (2017)	Treated cases only
Iran	1.4	Parandin *et al*. (2021)	Treated cases only
Morocco	0.5 (0.3–0.8)	Saadi *et al*. (2020)	Treated cases only, Discounted and age weighted
Peru	3.3 (2.5–4.3)	Moro *et al*. (2011)	Treated cases only
Qinghai-Tibet Plateau (China)	248 (240–269)	Wang *et al*. (2020)	Prevalence YLDs. Data from imaging surveillance studies
Sardinia (Italy)	30 (26–35)	Mastrandrea *et al*. (2012)	Treated cases only, Discounted and age weighted
Xingjiang (China)	2	Wang *et al*. (2012)	Only cases from 5 hospitals. Discounted and age weighte

### Strengths and limitations

This study expands upon the previous observations concerning the epidemiology of CE in Bhutan (Thapa *et al*., 2017; Kelzang *et al*., 2019; Sharma *et al*., 2021) by including records of human CE cases over a 5-year period from all districts recorded and managed at the JDWNRH. Given that no patients were operated on at the regional referral and other district-level hospitals in the given period (verbal communication with surgeons at the regional referral hospitals (*Dr S. Kelzang & Dr S. Jamtsho, personal communication, September 03, 2022)*, the patients reported and managed at JDWNRH are likely to represent most cases detected in the country of that period. Since the data was obtained from hospital records, the data collected was accurate and reliable for those treated patients. The findings provide a crude burden by districts and regions and the potential risk factors in Bhutan that can be used to inform initial public health policies.

Bhutan is a small lower middle income country with little engagement with the international scientific community. This report therefore provides important epidemiological data which demonstrates that echinococcosis is highly endemic in the country and indicates the extent of the health burden of a neglected tropical disease. Thus the information will be valuable to global public health organizations such as the World Health Organisation. Indeed, it is the only data on echinococcosis available from Bhutan that can be used in the WHO estimates of the burden of foodborne disease through the Foodborne Disease Burden Epidemiology Reference Group (FERG) initiative.

This study has some limitations. The burden is very likely to be underestimated since clinically diagnosed cases may represent only a trivial portion of the total number of actual cases. However, when estimating the DALYs we have illustrated this with an estimate for to include estimated numbers of undiagnosed cases. The absence of cases of the brain, heart, and bones also suggests potential under recording of cases. Although, validation through cross-checking of cases recorded in JDWNRH and those registered in the district hospitals may yield better estimates, this was not possible due to a lack of a standardized recording and reporting system. The US reports are currently not read by a blinded experienced person and is not classified using the WHO-IWG criteria, which might have affected the reliability of the diagnosis to some extent. Similarly, information on serological status was not available which otherwise could help in confirming the diagnosis. Some individuals might have also acquired infection during their stay at the present addresses, while the addresses that we obtained for this study were their permanent addresses.

Furthermore, information on some variables such as length of stay, treatment type, treatment outcome, geographic location of the case, and occupation was incomplete or not available. These recording deficiencies are expected since control of CE has not currently received much attention. The descriptive analysis used in this study only allows to make summations of the sample analysis. Multivariable analysis using a Poisson regression model failed to find any association of CE incidence with animal population density. Other multivariable analyses were not possible owing to data limitations. Given the small number of observations (cases), the parameter estimates for the case notification rates were estimated using the total cases reported in the five-year period, which might have overestimated the estimates to some extent. Nonetheless, this might have been offset by the potential underreporting of cases in hospital records discussed earlier. This study also could not assess other factors such as knowledge, health behaviour, eating habits, living environment, drinking water and sanitation, and management of dogs and domestic animals (Acosta-Jamett *et al*., 2014; Wang *et al*., 2014; Possenti *et al*., 2016; Conraths *et al*., 2017; Yuan *et al*., 2017; Cadavid Restrepo et al., 2018; Haleem *et al*., 2018), which is crucial in identifying high-risk groups and developing targeted policy interventions.

## Conclusions

Given that the majority of the population depends on livestock farming, especially in rural areas, a large section of the population is at risk for CE in Bhutan. A total of 159 cases were detected and recorded in the period 2015–2019. CE also contributes to the rising healthcare cost in Bhutan. The case notification rates were higher among females, those aged 30–59 years, and farmers suggesting that these groups are most affected. The central districts, mainly Bumthang, Wangdue Phodrang, Trongsa, and Paro, and Haa in the western region also had high case notification rates.

Policy interventions targeting farmers, females, older adults, and the high endemic districts of the central and western regions may yield larger returns. Initial activities may include health education for health workers, training of health workers in case detection and management, control of stray dog populations and deworming of dogs (both pet and stray), and the institution of a recording and reporting system that ought to be carried out in collaboration with the animal health and other relevant sectors through One Health approach. Future prospective studies assessing the burden, geographic distribution, and disparities, and examining a range of plausible risk factors are needed. Studies also need to use the WHO-IWGE criteria and recommended diagnostic approaches to define and/or classify cases. Such developments are essential for the national programme and related agencies to help develop effective interventions for the prevention and control of CE in Bhutan.

## Supporting information

Tenzin et al. supplementary material Tenzin et al. supplementary material

## Data Availability

All the data analysed is in the manuscript or supplementary material. Other information will be available on a reasonable request from the principal investigator.

## Supplementary material

The supplementary material for this article can be found at https://doi.org/10.1017/S0031182024001069.
