# Supplementary material for: The burden and distribution of cystic echinococcosis in Bhutan: a retrospective study
Source: Parasitology. 2024 Nov 8;151(13):1432–40. doi: 10.1017/S0031182024001069 (PMC12037339; doi:10.1017/S0031182024001069)
Supplement: Tenzin et al. supplementary material [file S0031182024001069sup001.pdf]

Supplementary Material R code and Output

Paul Torgerson

2024-06-04

```
{r setup, include=FALSE}
knitr::opts_chunk$set(echo = TRUE)
options(width=60)
```

```r
#Supplementary Material

library(dirmult)
library(MASS)
library(AICcmodavg)
library(glmmTMB)

#DALY Estimates

cses<-c(30, 35,32,26,24,12)
incidence<-rdirichlet(n=10000, alpha=cses)
pr<-rgamma(10000, 159, 5)
cases<-incidence*pr
View(cases)
quantile(cases[,1], c(0.025, 0.5, 0.975))
```

```
##     2.5%      50%    97.5%
## 4.063168 5.912168 8.313809
```

```r
cases1<-cases*0.02
lexp<-c(75.09,65.14,55.235,45.385,35.715,17.705)
ylls<-lexp*cases1
ylls1<-rowSums(ylls)
quantile(ylls1, c(0.025, 0.5, 0.975))
```

```
##     2.5%      50%    97.5%
## 23.07678 30.99971 40.38210
```

```r
yldliv<-0.78*2*rnorm(1,0.123,.02)*cases
yldpul<-0.13*2*rnorm(1,0.192, 0.032)*cases
yldcns<-0.07*2*rnorm(1, 0.221,0.04) *cases
ylds<-yldliv+yldpul+yldcns
DALYs<-ylds+ylls
DALYs1<-rowSums(DALYs)
quantile(DALYs1, c(0.025,0.5,0.975))
```

```
##     2.5%      50%    97.5%
## 31.86401 40.81948 51.36159
```

```r
#For untreated cases
untreatedDALYs<-pr*100*rnorm( 1, 0.012, 0.001)
quantile(untreatedDALYs, c(0.026,0.5,0.975))
```

```
##     2.6%      50%    97.5%
## 29.72457 34.78164 40.63422
```

```r
#Total DALYs
DALYs2<-DALYs1+untreatedDALYs
quantile(DALYs2, c(0.025,0.5,0.975))
```

```
##     2.5%      50%    97.5%
## 62.43830 75.70175 90.82092
```

```r
#per 100,000
DALYs3<-DALYs2*100000/782455
quantile(DALYs3, c(0.025,0.5,0.975))
```

```
##      2.5%       50%     97.5%
##  7.979795  9.674901 11.607175
```

```r
library(readxl)
# Read in data from excel file
bhu <- read_excel("bhu.xlsx", sheet = "Sheet1")

# data
bhu
```

```
## # A tibble: 6 x 9
##   district        cases   pop Cattle  Dogs  Yaks Sheep Goats  area
##   <chr>           <dbl> <dbl>  <dbl> <dbl> <dbl> <dbl> <dbl> <dbl>
## 1 Bumthang           36 17820  11311  1017  3917   127     9  2717
## 2 Punakha            16 28750  11045  1394     0   146   208  1110
## 3 Trongsa             6 19960  11583   544   226   132    92  1807
## 4 Zhemgang5           1 17763  12110   581     0     0    37  2421
## 5 WangduePhodrang    32 42186  23306  1152  4115   948    88  4308
## 6 Gasa                1  3952   1163   193  6527     0     2  3118
```

```r
#Forward selection Poisson regression
#data is overdispersed so generalized poisson model is used.
#Controlled for population size (offset variable)
res<-glmmTMB(cases~I(Cattle/area)+offset(log(pop)),
             family=genpois, data=bhu)
```

```
## Warning in (function (start, objective, gradient = NULL, hessian = NULL, : NA/
## NaN function evaluation
```

```r
AICc(res)
```

```
## [1] 59.16588
```

```r
#AICc=59.2

res<-glmmTMB(cases~I(Yaks/area)+offset(log(pop)),
             family=genpois, data=bhu)
#Yak density p=0.0519, so retained in model
AICc(res)
```

```
## [1] 56.28484
```

```r
#AICc=56.3

res<-glmmTMB(cases~I(Yaks/area)+I(Dogs/area)+offset(log(pop)),
             family=genpois, data=bhu)
```

```r
# Yak density and dog density retained
AICc(res)
```

```
## [1] 84.09494
```

```r
# AICc = 84.1

res<-glmmTMB(cases~I(Yaks/area)+I(Dogs/area)+I(Sheep/area)+offset(log(pop)),
             family=genpois, data=bhu)
# Sheep density removed from model
AICc(res)
```

```
## [1] Inf
```

```r
# overfitted; AICc inf

res<-glmmTMB(cases~I(Dogs/area)+offset(log(pop)),family=genpois, data=bhu)
AICc(res)
```

```
## [1] 59.09987
```

```r
# Dog densisty
# AICc = 59.1

res<-glmmTMB(cases~I(Yaks/area)+I(Dogs/area)+I(Goats/area)+offset(log(pop)),
             family=genpois, data=bhu)
AICc(res)
```

```
## [1] Inf
```

```r
#over fitted, AICc inf

res<-glmmTMB(cases~offset(log(pop)),family=genpois, data=bhu)
```

```
## Warning in (function (start, objective, gradient = NULL, hessian = NULL, : NA/
## NaN function evaluation

## Warning in (function (start, objective, gradient = NULL, hessian = NULL, : NA/
## NaN function evaluation

## Warning in (function (start, objective, gradient = NULL, hessian = NULL, : NA/
## NaN function evaluation

## Warning in (function (start, objective, gradient = NULL, hessian = NULL, : NA/
## NaN function evaluation

## Warning in (function (start, objective, gradient = NULL, hessian = NULL, : NA/
## NaN function evaluation

## Warning in (function (start, objective, gradient = NULL, hessian = NULL, : NA/
## NaN function evaluation
```

```r
AICc(res)
```

```
## [1] 49.19919
```

```r
# null model
# AICc=49.2
```

```r
res<-glmmTMB(cases~I(Sheep/area)+offset(log(pop)),
             family=genpois, data=bhu)
```

```
## Warning in (function (start, objective, gradient = NULL, hessian = NULL, : NA/
## NaN function evaluation
```

```r
AICc(res)
```

```
## [1] 58.24506
```

```r
# model sheep only
#AICc = 58.24506

# All models with covariates had a higher AICc than null model,
#which suggests that there is insufficient evidence to conclude
#that density of livestock or dogs is associated with the incidence of echinococcosis.

#Likewise using the negative binomial model as alternative overdispersed model

# null model
res<-glm.nb(cases~offset(log(pop)), data=bhu)
AICc(res)
```

```
## [1] 50.08705
```

```r
summary(res)
```

```
##
## Call:
## glm.nb(formula = cases ~ offset(log(pop)), data = bhu, init.theta = 1.269242805,
##     link = log)
##
## Coefficients:
##             Estimate Std. Error z value Pr(>|z|)
## (Intercept)  -7.3013     0.3857  -18.93   <2e-16 ***
## ---
## Signif. codes:  0 '***' 0.001 '**' 0.01 '*' 0.05 '.' 0.1 ' ' 1
##
## (Dispersion parameter for Negative Binomial(1.2692) family taken to be 1)
##
##     Null deviance: 6.3245  on 5  degrees of freedom
## Residual deviance: 6.3245  on 5  degrees of freedom
## AIC: 46.087
##
## Number of Fisher Scoring iterations: 1
##
##
##               Theta:  1.269
##           Std. Err.:  0.799
##
##  2 x log-likelihood:  -42.087
```

```r
#lowest AICc
#Conclude no significant explanatory variables

# dogs
res<-glm.nb(cases~I(Dogs/area)+(offset(log(pop))), data=bhu)
```

```r
AICc(res)
```

```
## [1] 60.05561
```

```r
summary(res)
```

```
##
## Call:
## glm.nb(formula = cases ~ I(Dogs/area) + (offset(log(pop))), data = bhu,
##     init.theta = 1.270574786, link = log)
##
## Coefficients:
##              Estimate Std. Error z value Pr(>|z|)
## (Intercept)   -7.3913     0.5794 -12.756   <2e-16 ***
## I(Dogs/area)   0.2050     0.9851   0.208    0.835
## ---
## Signif. codes:  0 '***' 0.001 '**' 0.01 '*' 0.05 '.' 0.1 ' ' 1
##
## (Dispersion parameter for Negative Binomial(1.2706) family taken to be 1)
##
##     Null deviance: 6.3300  on 5  degrees of freedom
## Residual deviance: 6.2986  on 4  degrees of freedom
## AIC: 48.056
##
## Number of Fisher Scoring iterations: 1
##
##
##               Theta:  1.271
##           Std. Err.:  0.798
##
##  2 x log-likelihood:  -42.056
```

```r
#etc with other animal species.

#Alternatively random effects model with district as the random effect.
#Models overdispersion by the random effect
res<-glmmTMB(cases~I(Dogs/area)+(offset(log(pop))+(1|district)),
             poisson, data=bhu)
summary(res)
```

```
##  Family: poisson  ( log )
## Formula:          cases ~ I(Dogs/area) + (offset(log(pop)) + (1 | district))
## Data: bhu
##
##      AIC      BIC   logLik deviance df.resid
##     47.9     47.3    -20.9     41.9        3
##
## Random effects:
##
## Conditional model:
##  Groups   Name        Variance Std.Dev.
##  district (Intercept) 0.8742   0.935
## Number of obs: 6, groups:  district, 6
##
## Conditional model:
```

```
##              Estimate Std. Error z value Pr(>|z|)
## (Intercept)   -7.9601     0.6695 -11.890   <2e-16 ***
## I(Dogs/area)   0.5553     1.0786   0.515    0.607
## ---
## Signif. codes:  0 '***' 0.001 '**' 0.01 '*' 0.05 '.' 0.1 ' ' 1
```

```r
AICc(res)
```

```
## [1] 59.89844
```

```r
res<-glmmTMB(cases~I(Yaks/area)+(offset(log(pop))+(1|district)),
             poisson, data=bhu)
summary(res)
```

```
##  Family: poisson  ( log )
## Formula:          cases ~ I(Yaks/area) + (offset(log(pop)) + (1 | district))
## Data: bhu
##
##      AIC      BIC   logLik deviance df.resid
##     46.7     46.1    -20.4     40.7        3
##
## Random effects:
##
## Conditional model:
##  Groups   Name        Variance Std.Dev.
##  district (Intercept) 0.5251   0.7246
## Number of obs: 6, groups:  district, 6
##
## Conditional model:
##              Estimate Std. Error z value Pr(>|z|)
## (Intercept)   -8.1010     0.4866 -16.649   <2e-16 ***
## I(Yaks/area)   0.6379     0.4553   1.401    0.161
## ---
## Signif. codes:  0 '***' 0.001 '**' 0.01 '*' 0.05 '.' 0.1 ' ' 1
```

```r
AICc(res)
```

```
## [1] 58.71032
```

```r
res<-glmmTMB(cases~I(Cattle/area)+(offset(log(pop))+(1|district)),
             poisson, data=bhu)
summary(res)
```

```
##  Family: poisson  ( log )
## Formula:          cases ~ I(Cattle/area) + (offset(log(pop)) + (1 | district))
## Data: bhu
##
##      AIC      BIC   logLik deviance df.resid
##     48.1     47.5    -21.1     42.1        3
##
## Random effects:
##
## Conditional model:
##  Groups   Name        Variance Std.Dev.
##  district (Intercept) 0.8975   0.9473
## Number of obs: 6, groups:  district, 6
##
```

```
## Conditional model:
##                Estimate Std. Error z value Pr(>|z|)
## (Intercept)    -7.89546    1.02968  -7.668 1.75e-14 ***
## I(Cattle/area)  0.03311    0.16295   0.203    0.839
## ---
## Signif. codes:  0 '***' 0.001 '**' 0.01 '*' 0.05 '.' 0.1 ' ' 1
```

```
AICc(res)
```

```
## [1] 60.12713
```

```
res<-glmmTMB(cases~I(Sheep/area)+(offset(log(pop))+(1|district)),
             poisson, data=bhu)
summary(res)
```

```
##  Family: poisson  ( log )
## Formula:          cases ~ I(Sheep/area) + (offset(log(pop)) + (1 | district))
## Data: bhu
##
##      AIC      BIC   logLik deviance df.resid
##     47.1     46.4    -20.5     41.1        3
##
## Random effects:
##
## Conditional model:
##  Groups   Name        Variance Std.Dev.
##  district (Intercept) 0.8004   0.8946
## Number of obs: 6, groups:  district, 6
##
## Conditional model:
##               Estimate Std. Error z value Pr(>|z|)
## (Intercept)    -8.2061     0.6654 -12.333   <2e-16 ***
## I(Sheep/area)   5.6092     5.4152   1.036      0.3
## ---
## Signif. codes:  0 '***' 0.001 '**' 0.01 '*' 0.05 '.' 0.1 ' ' 1
```

```
AICc(res)
```

```
## [1] 59.0517
```

```
res<-glmmTMB(cases~I(Goats/area)+(offset(log(pop))+(1|district)),
             poisson, data=bhu)
summary(res)
```

```
##  Family: poisson  ( log )
## Formula:          cases ~ I(Goats/area) + (offset(log(pop)) + (1 | district))
## Data: bhu
##
##      AIC      BIC   logLik deviance df.resid
##     48.2     47.5    -21.1     42.2        3
##
## Random effects:
##
## Conditional model:
##  Groups   Name        Variance Std.Dev.
##  district (Intercept) 0.8875   0.9421
## Number of obs: 6, groups:  district, 6
```

```
##
## Conditional model:
##                 Estimate Std. Error z value Pr(>|z|)
## (Intercept)      -7.7237     0.5540 -13.942   <2e-16 ***
## I(Goats/area)     0.2979     6.3244   0.047    0.962
## ---
## Signif. codes:  0 '***' 0.001 '**' 0.01 '*' 0.05 '.' 0.1 ' ' 1
```

```r
AICc(res)
```

```
## [1] 60.16706
```

```r
res<-glmmTMB(cases~I(Goats/area)+(offset(log(pop))+(1|district)),
             poisson, data=bhu)
summary(res)
```

```
##  Family: poisson  ( log )
## Formula:          cases ~ I(Goats/area) + (offset(log(pop)) + (1 | district))
## Data: bhu
##
##      AIC      BIC   logLik deviance df.resid
##     48.2     47.5    -21.1     42.2        3
##
## Random effects:
##
## Conditional model:
##  Groups   Name        Variance Std.Dev.
##  district (Intercept) 0.8875   0.9421
## Number of obs: 6, groups:  district, 6
##
## Conditional model:
##                 Estimate Std. Error z value Pr(>|z|)
## (Intercept)      -7.7237     0.5540 -13.942   <2e-16 ***
## I(Goats/area)     0.2979     6.3244   0.047    0.962
## ---
## Signif. codes:  0 '***' 0.001 '**' 0.01 '*' 0.05 '.' 0.1 ' ' 1
```

```r
AICc(res)
```

```
## [1] 60.16706
```

```r
# Null random effects model with intercept only
res<-glmmTMB(cases~(offset(log(pop))+(1|district)),
             poisson, data=bhu)
AICc(res)
```

```
## [1] 50.16929
```

```r
#lowest AICc
#Conclude no significant explanatory variables
```
